# Compact large language models for title and abstract screening in systematic reviews: An assessment of feasibility, accuracy, and workload reduction

**DOI:** 10.1017/rsm.2025.10044

**Published:** 2025-11-13

**Authors:** Antonio Sciurti, Giuseppe Migliara, Leonardo Maria Siena, Claudia Isonne, Maria Roberta De Blasiis, Alessandra Sinopoli, Jessica Iera, Carolina Marzuillo, Corrado De Vito, Paolo Villari, Valentina Baccolini

**Affiliations:** 1 Department of Public Health and Infectious Diseases, University of Rome La Sapienza, Italy; 2 Department of Life Sciences, Health, and Health Professions, Link Campus University, Italy; 3 Department of Prevention, Local Health Authority Rome 1, Italy; 4 Department of Infectious Diseases, Istituto Superiore di Sanità, Italy

**Keywords:** artificial intelligence, Gemma 2 9B, GPT-4o mini, large language models, Llama 3.1 8B, title and abstract screening

## Abstract

Systematic reviews play a critical role in evidence-based research but are labor-intensive, especially during title and abstract screening. Compact large language models (LLMs) offer potential to automate this process, balancing time/cost requirements and accuracy. The aim of this study is to assess the feasibility, accuracy, and workload reduction by three compact LLMs (GPT-4o mini, Llama 3.1 8B, and Gemma 2 9B) in screening titles and abstracts. Records were sourced from three previously published systematic reviews and LLMs were requested to rate each record from 0 to 100 for inclusion, using a structured prompt. Predefined 25-, 50-, 75-rating thresholds were used to compute performance metrics (balanced accuracy, sensitivity, specificity, positive and negative predictive value, and workload-saving). Processing time and costs were registered. Across the systematic reviews, LLMs achieved high sensitivity (up to 100%) and low precision (below 10%) for records included by full text. Specificity and workload savings improved at higher thresholds, with the 50- and 75-rating thresholds offering optimal trade-offs. GPT-4o-mini, accessed via application programming interface, was the fastest model (~40 minutes max.) and had usage costs ($0.14–$1.93 per review). Llama 3.1-8B and Gemma 2-9B were run locally in longer times (~4 hours max.) and were free to use. LLMs were highly sensitive tools for the title/abstract screening process. High specificity values were reached, allowing for significant workload savings, at reasonable costs and processing time. Conversely, we found them to be imprecise. However, high sensitivity and workload reduction are key factors for their usage in the title/abstract screening phase of systematic reviews.

## Highlights

### What is already known?

- Large language models (LLMs) have shown potential to automate the title/abstract screening process of systematic reviews, but practical aspects of their usage, such as costs and processing time, should be considered.

### What is new?

- Compact LLMs can achieve high sensitivity and substantial workload reduction in the title/abstract screening of different reviews, with reasonable costs and processing time.

### Potential impact for RSM readers

- This study provides systematic review authors with a practical, reproducible approach to integrating compact LLMs into title and abstract screening for their own reviews.

## 1 Introduction

Systematic reviews are a cornerstone in evidence-based research, offering comprehensive insights into complex questions, although they demand significant time and effort.^1^ It was estimated that completing a systematic review may require 67.3 weeks and about 5.3 team members on average.^2^ In particular, the selection of relevant articles is a key step of the systematic review workflow, yet it is time-consuming and labor-intensive.^3^ It is a two-stage process that requires two reviewers or more: based on predefined inclusion and exclusion criteria, reviewers first screen titles and abstracts of the retrieved records, and then assess the full texts of the selected records.^3^ Despite the exhaustive process, usually only a small fraction of articles are included in the end.^2^

Artificial intelligence (AI) and machine learning (ML) have emerged as potential solutions to reduce this workload.^4^ Traditional ML approaches have shown promise in the title/abstract screening, but these tools rely heavily on human-labeled data and still require significant manual effort, limiting their scalability and generalizability.^5^ ^,^ ^6^ In contrast, large language models (LLMs) hold the potential to radically change the systematic review automation scenario.^7^ Thanks to self-attention mechanism-based architectures and pretraining on vast datasets,^8^ these models allow for conversational interactions with users and excel at natural language processing (NLP) tasks, such as text annotation,^9^ ^,^ ^10^ and can screen articles without additional training, achieving comparable or superior performance to traditional ML methods.^11^ ^–^ ^13^ Research on their application in systematic reviews is rapidly expanding, with most studies focusing on various versions of Generative Pretrained Transformer (GPT) developed by OpenAI^11^ ^–^ ^15^ and others including open source models as well.^16^ ^,^ ^17^

However, as most of these LLMs incur usage costs and demand substantial computational resources, recent advances, such as quantization, pruning, and distillation techniques, have led to the development of compact LLMs, also called small language models (SLMs), which balance performance with reduced costs and computational resource requirements.^18^ ^–^ ^20^ There is no universally accepted definition of such reduced models, although some operational definitions have been proposed, mostly based on the number of the model’s parameters (i.e., the weights and biases that a model learns in its training and ultimately determine the model’s complexity), with models under 10 billion parameters typically considered as compact LLMs.^21^ These lightweight models may offer an opportunity to reduce the workload of the title and abstract screening phase of systematic reviews in a cost-efficient way, while maintaining reasonable accuracy. While published studies concentrated on conventional LLMs’ screening performance,^11^ ^,^ ^12^ ^,^ ^15^ ^–^ ^17^ to the best of our knowledge, compact LLMs have not been explored yet in this context and, a comprehensive assessment of performance, time efficiency, and costs for their usage is lacking. Therefore, this study aims to assess the performance, required time, and costs of three compact LLMs, GPT-4o mini, Llama 3.1 8B, and Gemma 2 9B, in screening titles and abstracts from three previously published systematic reviews.

## 2 Methods

### 2.1 Data collection, prompt engineering, and interaction with LLMs

Records were sourced from three previously published systematic reviews. In brief, the first systematic review explored the association between vaccine literacy and vaccination intention/status (VL, hereinafter),^22^ while the second review investigated the impact of antibiotic exposure on antibiotic-resistant *Acinetobacter baumannii* isolation (AB, hereinafter).^23^ The third systematic review examined the efficacy of vitamin supplements in managing and preventing COVID-19 (COVID-19, hereinafter).^24^ Each record was originally screened by title/abstract, and subsequently by full-text and manually labeled as included or excluded by two authors. Disagreements were resolved by a third author. Residual duplicate records were removed from the AB and COVID-19 reviews.

Each record’s title and abstract were embedded into a structured prompt. The prompt engineering strategy was based on structuring prompts into three main components, as proposed by Syriani et al.^11^ ^,^ ^12^—(i) “context”, (ii) “instructions”, and (iii) “task”:

- “Context” provided general information about the systematic review topic using a *persona* approach.^25^
- “Instructions” detailed screening criteria for determining inclusion or exclusion based on the title and abstract. The instructions were to rate each record from 0 (least confident) to 100 (most confident), based on inclusion confidence. Both context and instructions were tailored for each systematic review. A zero-shot prompting approach was employed, that is, examples of included and excluded records were not provided, and exclusion criteria were avoided to prioritize sensitivity.^12^ ^,^ ^26^
- “Task” included the title and abstract of the record to be screened.

Examples of used prompts are shown in Table S1 in the Supplementary Material.

The three LLMs were queried using the structured prompts for each record and systematic review. GPT-4o mini is a proprietary model by OpenAI, which involves usage costs as it operates through OpenAI’s application programming interface (API), with its exact number of parameters remaining undisclosed.^27^ It was accessed via OpenAI’s API using the *oaii* R package (ver. 0.5.0)^28^ (GPT-4o mini ver. 2024-07-18, last date of training October 2023).^29^ In contrast, Llama 3.1 8B and Gemma 2 9B are open-source models, with 8 and 9 billion parameters and developed by Meta and Google, respectively, which can be downloaded and run on local machines, with processing times heavily dependent on the hardware capabilities.^30^ ^,^ ^31^ Llama 3.1 8B (id. 365c0bd3c000, last date of training December 2023)^32^ and Gemma 2 9B (id. ff02c3702f32, last date of training not disclosed)^33^ were accessed locally via the Ollama application (ver. 0.5.1),^34^ using the *rollama* R package (ver. 0.2.0).^35^

The “context” and “instructions” components of each prompt were supplied to the models as the *system* role, and the “task” component as the *user* role.^9^ Model hyperparameters were standardized across models. *Temperature* ranges from 0 to 1 and controls the diversity of the model’s responses,^36^ although a deterministic output is not guaranteed.^37^ Therefore, we set the same random *seed* and a *temperature* of 0 to maximize reproducibility. The maximum number of output tokens (*max_tokens* or *num_predict*) determines the length of the model’s responses.^28^ ^,^ ^35^ It was set to 1 to restrict the amount of generated text to the required responses, thereby reducing costs and time to responses. As the model outputs were provided as strings, the responses were converted to their corresponding integers. Invalid responses, that is, those different from a number between 0 and 100, were registered and set to 0. Records without abstract were not excluded. If a records’ abstract was missing, only the title was used in the structured prompt. Finally, responses per minute (that is, the number of requests made to the LLM in a minute), overall time to responses (that is the overall time needed to screen records), and overall costs were recorded.

### 2.2 Statistical analysis

**Figure 1:**
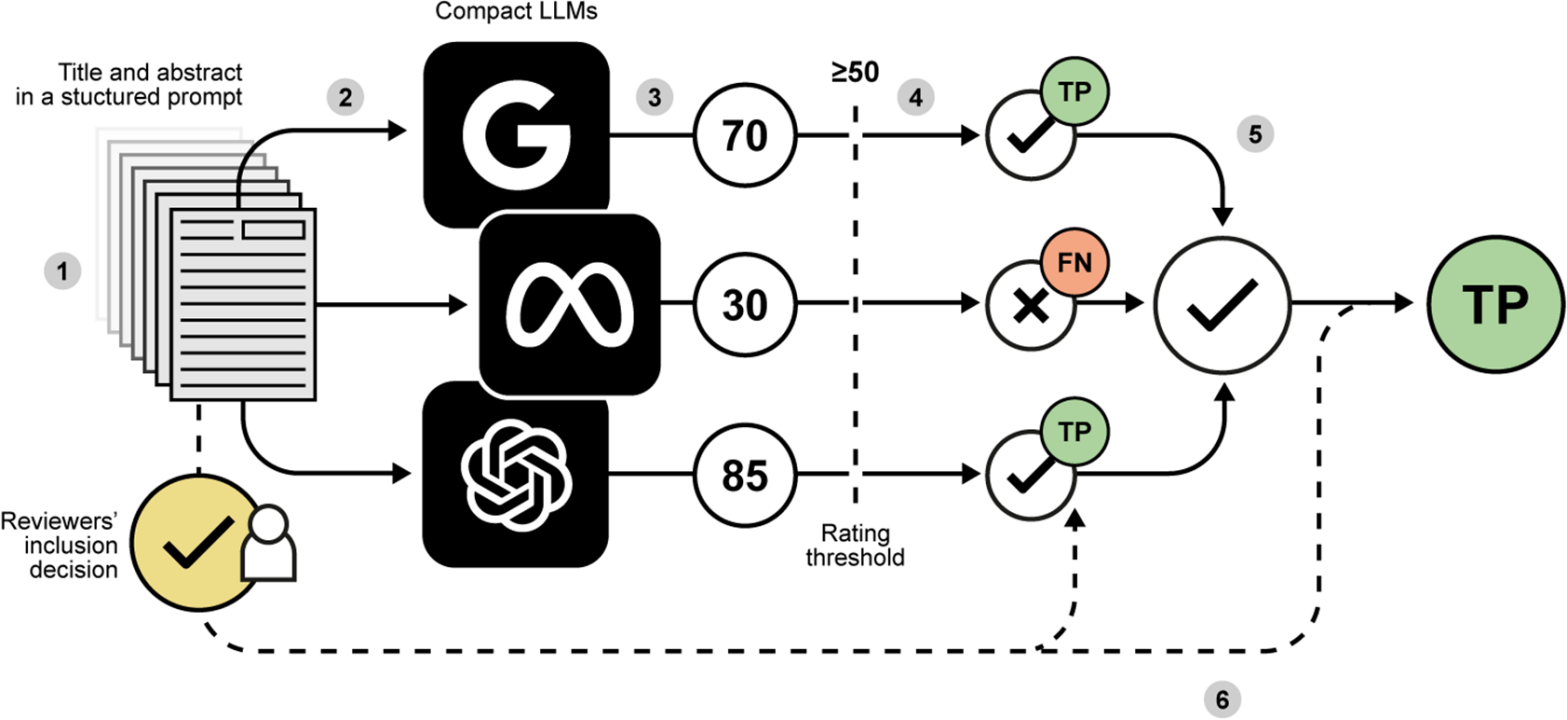
Visual example of the inclusion decision process for a single record within each systematic review. (1) The record’s title and abstract are embedded into a structured prompt; (2) The prompt is fed into each of the three LLMs; (3) Each LLM rates the record with an integer number from 0 to 100, according to the prompt; (4) If rating meets or exceeds the threshold, record is included (individual LLM decision, ✓: included, ×: excluded); (5) Individual LLM decisions are combined through majority voting; (6) Individual LLM decisions and majority voting are compared with the reviewers’ decision (TP: true positive; TN: true negative; FP: false positive; FN: false negative), for performance assessment.

Performance metrics were calculated using predefined inclusion thresholds at the 25-, 50-, and 75-rating, for each of the three models and estimated against both the original author-labeled screening by title/abstract as the reference standard. In addition, for each of the three predefined thresholds, inclusion decisions by each model were combined using a majority voting ensemble strategy, where the final inclusion decision is based on the majority, i.e., the most frequent, decision of the three models.^38^ Moreover, we used original author-labeled screening by full-text as an additional reference standard for a sensitivity analysis.^39^ This aimed to verify whether all relevant articles would be included by the models regardless of their performance on title and abstract screening, as truly relevant articles are those included after full-text screening. An example of the entire inclusion process is shown in Figure 1.

As suggested by Syriani et al.^11^ performance metrics included sensitivity, specificity, positive predictive value (PPV), negative predictive value (NPV), balanced accuracy, and workload saving. Sensitivity (sometimes referred to as “recall”) measures an LLM’s ability to include all records that should be included, while specificity expresses the model’s ability to exclude all records that should be excluded. Conversely, PPV (sometimes referred to as “precision”) shows a model’s ability to include only articles that should be included and NPV a models’ ability to exclude only articles that should be excluded. Balanced accuracy, calculated as the arithmetic mean of sensitivity and specificity, is an overall accuracy metric well suited for assessing imbalanced class situations—the common scenario in the screening of records in systematic reviews.^2^ ^,^ ^26^ Finally, workload saving is nonstandard metric to evaluate workload reduction by automated screening tools, expressing records correctly excluded by the model, out of the total number of screened records.^11^ A formal description of performance metrics is provided in Table S2 in the Supplementary Material. The *caret* R package (ver. 7.0.1)^40^ was used to compute the performance metrics.

As a supplementary analysis, Receiver Operating Characteristic (ROC) curves were plotted, and the area under the curve (AUC) was calculated for each model, using the *pROC* R package (ver. 1.18.5).^41^ With records assigned a number ranging from 0 to 100, thresholds for inclusion could be defined. Optimal thresholds for inclusion were determined according to the Closest Top-Left method, and performance metrics were computed using the selected thresholds.

All computations were run on a 13^th^ Gen Intel® Core™ i9-13900K 3.00 GHz CPU with 64 GB RAM and a NVIDIA RTX A2000 12GB RAM GPU, on a 64-bit Windows 11 Pro system. All analyses were performed using R Statistical Software (version 4.4.2; R Core Team 2024, R Foundation for Statistical Computing, Vienna, Austria). Datasets and R code are available on Open Science Framework (OSF) (https://osf.io/kjnwt).^42^

This study was reported according to the TRIPOD + LLM reporting guidelines for studies evaluating LLMs in classification tasks (Table S3 in the Supplementary Material).^43^

## 3 Results

### 3.1 Characteristics of systematic reviews

The characteristics of the systematic reviews are presented in Table 1. The VL systematic review screened 1,757 records by title and abstract, published between 1976 and 2022. In all, 64 records (3.6%) were included after title/abstract screening, and 18 (1.0%) were included after full-text screening. The AB systematic review had the largest number of records screened, 21,116 (published between 1956 and 2023), of which 322 (1.5%) were included after screening by title and abstract, and 25 (0.1%) after the screening by full-text. In the COVID-19 systematic review, 7,693 records were screened by title and abstract (published between 1985 and 2024), 72 (0.9%) of which were included after title/abstract screening, and 37 (0.5%) after full-text screening. The three systematic reviews had 8.6% (VL), 7.3% (AB), and 14.3% (COVID-19) records with a missing abstract.

**Table 1:** Characteristics of systematic reviews

					Records	Records	
				Record	included by	included by	
			Overall	publication	title/abstract	full-text	Records with
Systematic			records	time range,	screening, *N*	screening, *N*	missing
review	Author, year	Title	screened, *N*	years	(%)	(%)	abstract, *N* (%)
VL	Isonne et al., 2024^22^	“How well does vaccine literacy predict intention to vaccinate and vaccination status? A systematic review and meta-analysis”	1,757	1976–2022	64 (3.6)	18 (1.0)	151 (8.6)
AB	De Blasiis et al., 2024^23^	“Impact of antibiotic exposure on antibiotic-resistant *Acinetobacter baumannii* isolation in intensive care unit patients: a systematic review and meta-analysis”	21,116	1956–2023	322 (1.5)	25 (0.1)	1,544 (7.3)
COVID-19	Sinopoli et al., 2024^24^	“The efficacy of multivitamin, vitamin A, vitamin B, vitamin C, and vitamin D supplements in the prevention and management of COVID–19 and long-COVID: an updated systematic review and meta-analysis of randomized clinical trials”	7,693	1985–2024	72 (0.9)	37 (0.5)	1,100 (14.3)

*Note:* VL: vaccine literacy; AB: *A. baumannii*; COVID-19: coronavirus disease 2019.

### 3.2 Performance metrics

Performance metrics computed based on 25-, 50-, and 75-rating thresholds and majority voting of models are shown in Table 2. When using the title/abstract screening as a reference, in the VL review the 75-rating threshold provided the highest balanced accuracy for the Llama 3.1 8B and Gemma 2 9B models (84.1% and 86.0%, respectively), while GPT-4o mini had the highest balanced accuracy using the 50-rating threshold (87.0%). Both the 25- and 50-rating thresholds yielded sensitivity above 90%, while specificities and workload savings were above 80% with the 75-rating threshold. In the AB review, the 50-rating threshold provided the highest balanced accuracy values for the GPT-4o mini (82.4%) and Gemma 2 9B (84.8%) models, with higher sensitivities (74.5% and 89.8%, respectively), but lower specificities (90.3% and 79.9%, respectively) and workload savings (89.0% and 78.7%, respectively), compared to the 75-rating threshold. In contrast, Llama 3.1 8B had consistently 56.2% balanced accuracy, 99.7% sensitivity, and around 12.7% specificity and workload savings across all three thresholds. In the COVID-19 review, the 50-rating threshold achieved the highest balanced accuracy for all the models (90.1% GPT-4o mini, 91.2% Llama 3.1 8B, and 88.9% Gemma 2 9 B), compared to the other thresholds. Sensitivities ranged between 87.5% and 93.1% using both 25- and 50-rating thresholds, and between 73.6% and 91.7% using the 75-rating threshold. Specificities, instead, were the highest, all above 90%, using the 75-rating threshold, with workload savings following a similar pattern. PPVs were lower than 10% with a 25-rating threshold across all systematic reviews and did not exceed 16% and 26% with a 50- and 75-rating threshold, respectively, while NPVs remained consistently around 99%. In general, the majority voting approach using a 50-rating threshold achieved performance comparable or better than the individual models, with balanced accuracies above 80%, sensitivities exceeding 90%, and specificities ranging from 68.3% and 91.7%. Similarly, using a 50-rating threshold, workload savings ranged between 65.9% and 90.8%.

**Table 2:** LLM performance metrics, expressed as percentage (%), by systematic review

Records screened by title/abstract used as a reference																			
		25-rating threshold						50-rating threshold						75-rating threshold					
Systematic review	LLM	bAcc	Sens	Spec	PPV	NPV	WS	bAcc	Sens	Spec	PPV	NPV	WS	bAcc	Sens	Spec	PPV	NPV	WS
VL	GPT-4o mini	80.3	96.9	63.8	9.2	99.8	61.5	87.0	93.8	80.3	15.3	99.7	77.4	84.7	78.1	91.3	25.4	99.1	88.0
	Llama 3.1 8B	81.5	96.9	66.0	9.7	99.8	63.6	81.5	96.9	66.0	9.7	99.8	63.6	84.1	82.8	85.4	17.7	99.2	82.3
	Gemma 2 9B	64.4	93.8	35.1	5.2	99.3	33.9	71.3	93.8	48.8	6.5	99.5	47.1	86.0	87.5	84.6	17.7	99.4	81.5
	Majority voting	78.2	98.4	57.9	8.1	99.9	55.8	82.6	96.9	68.3	10.4	99.8	65.9	86.3	84.4	88.3	21.4	99.3	85.1
AB	GPT-4o mini	76.3	98.8	53.8	3.2	100.0	53.0	82.4	74.5	90.3	10.7	99.6	89.0	81.3	71.4	91.1	11.0	99.5	89.7
	Llama 3.1 8B	56.2	99.7	12.7	1.7	100.0	12.5	56.2	99.7	12.7	1.7	100.0	12.5	56.2	99.7	12.8	1.7	100.0	12.6
	Gemma 2 9B	83.3	93.2	73.4	5.2	99.9	72.3	84.8	89.8	79.9	6.5	99.8	78.7	75.1	54.3	95.8	16.8	99.3	94.4
	Majority voting	76.1	99.4	52.8	3.2	100.0	52.0	84.7	90.4	78.9	6.2	99.8	77.7	81.8	73.3	90.3	10.5	99.5	88.9
COVID-19	GPT-4o mini	86.5	87.5	85.5	5.4	99.9	84.7	90.1	87.5	92.7	10.2	99.9	91.8	89.4	81.9	96.9	20.2	99.8	96.0
	Llama 3.1 8B	91.2	93.1	89.3	7.6	99.9	88.5	91.2	93.1	89.3	7.6	99.9	88.5	90.8	91.7	90.0	8.0	99.9	89.2
	Gemma 2 9B	86.1	91.7	80.4	4.2	99.9	79.7	88.6	88.9	88.3	6.7	99.9	87.4	85.0	73.6	96.4	16.0	99.7	95.5
	Majority voting	92.1	97.2	86.9	6.5	100.0	86.1	93.1	94.4	91.7	9.7	99.9	90.8	90.6	84.7	96.4	18.2	99.9	95.5
		Records screened by full-text used as a reference																	
		25-rating threshold						50-rating threshold						75-rating threshold					
Systematic review	LLM	bAcc	Sens	Spec	PPV	NPV	WS	bAcc	Sens	Spec	PPV	NPV	WS	bAcc	Sens	Spec	PPV	NPV	WS
VL	GPT-4o mini	81.1	100.0	62.2	2.7	100.0	61.6	89.2	100.0	78.4	4.6	100.0	77.6	94.9	100.0	89.7	9.1	100.0	88.8
	Llama 3.1 8B	82.2	100.0	64.4	2.8	100.0	63.7	82.2	100.0	64.4	2.8	100.0	63.7	89.1	94.4	83.7	5.7	99.9	82.9
	Gemma 2 9B	61.6	88.9	34.3	1.4	99.7	34.0	68.3	88.9	47.7	1.7	99.8	47.2	85.8	88.9	82.7	5.0	99.9	81.8
	Majority voting	78.2	100.0	56.4	2.3	100.0	55.8	83.3	100.0	66.6	3.0	100.0	66.0	93.3	100.0	86.5	7.1	100.0	85.7
AB	GPT-4o mini	76.5	100.0	53.1	0.3	100.0	53.0	92.7	96.0	89.5	1.1	100.0	89.3	93.1	96.0	90.2	1.2	100.0	90.1
	Llama 3.1 8B	56.3	100.0	12.5	0.1	100.0	12.5	56.3	100.0	12.5	0.1	100.0	12.5	56.3	100.0	12.6	0.1	100.0	12.6
	Gemma 2 9B	86.3	100.0	72.5	0.4	100.0	72.4	89.5	100.0	78.9	0.6	100.0	78.8	95.6	96.0	95.2	2.3	100.0	95.1
	Majority voting	76.1	100.0	52.1	0.2	100.0	52.0	89.0	100.0	78.0	0.5	100.0	77.9	92.7	96.0	89.4	1.1	100.0	89.3
COVID-19	GPT-4o mini	91.3	97.3	85.2	3.1	100.0	84.8	94.8	97.3	92.4	5.8	100.0	92.0	97.0	97.3	96.7	12.3	100.0	96.2
	Llama 3.1 8B	94.5	100.0	89.0	4.2	100.0	88.5	94.5	100.0	89.0	4.2	100.0	88.5	94.8	100.0	89.7	4.5	100.0	89.2
	Gemma 2 9B	87.4	94.6	80.1	2.2	100.0	79.7	91.3	94.6	87.9	3.7	100.0	87.5	94.0	91.9	96.1	10.3	100.0	95.7
	Majority voting	93.3	100.0	86.5	3.5	100.0	86.1	95.7	100.0	91.3	5.3	100.0	90.9	98.1	100.0	96.1	11.0	100.0	95.6

*Note*: 25-, 50- and 75-ratings were used as thresholds.

LLM: large language model; bAcc: balanced accuracy; Sens: sensitivity; Spec: specificity; PPV: positive predictive value; NPV: negative predictive value; WS: workload saving; VL: vaccine literacy; AB: *A. baumannii*; COVID-19: coronavirus disease 2019; GPT: generative pretrained transformer.

When using records screened by full-text as a reference, balanced accuracy values were similar to the values observed with the title/abstract screening as a reference for the 25-rating threshold in all reviews, and in general slightly higher with a 50- and 75-rating threshold. Notably, all the three thresholds reached a 100% sensitivity for at least one LLM across the three reviews, with the 50- and 75-rating thresholds having overall higher specificities and workload savings. PPVs were consistently below 5% for the 25- and 50-rating thresholds, and did not exceed 11% with a 75-rating threshold. In detail, at 100% sensitivity, workload saving ranged between 12.5% and 88.5%, at a 25- and 50- rating threshold, and between 12.6% and 89.2% at a 75-rating threshold, while PPV ranged between 0.1% and 4.2%, at a 25- and 50- rating threshold, and between 0.1% and 9.1% at a 75-rating threshold. In this scenario, the majority voting approach yielded perfect sensitivity for all of the reviews and reached specificities between 52.1% and 86.5% using the 25-rating threshold, between 66.6% and 91.3% using the 50-rating threshold, and between 86.5% and 96.1% using the 75-rating threshold. Workload savings showed a similar pattern.

### 3.3 Optimal rating threshold analysis

**Figure 2:**
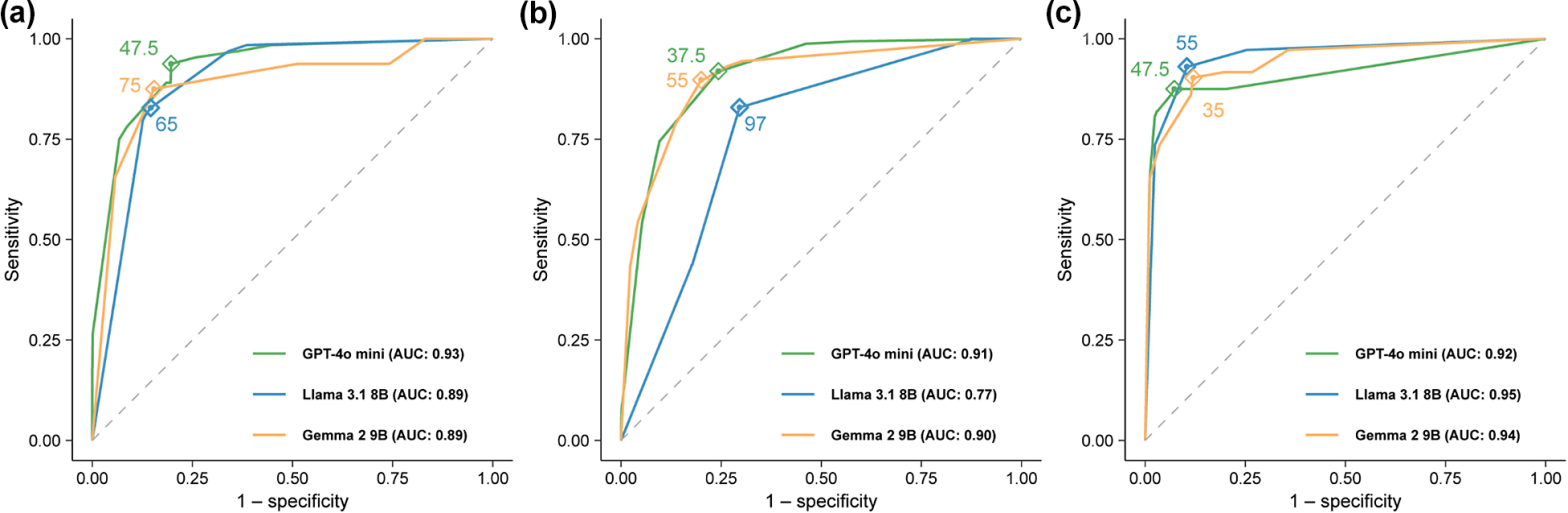
LLMs ratings ROC curves, by systematic review. (a) VL; (b) AB; (c) COVID-19. *Note*: LLM: large language model; VL: vaccine literacy; AB: Acinetobacter baumannii; COVID-19: coronavirus disease 2019; AUC: area under the curve; GPT: generative pretrained transformer.

Overall, AUCs over 0.75 were reached across different systematic reviews (Figure 2). Optimal rating thresholds varied by model and review, with GPT-4o mini showing a 47.5-rating optimal threshold for the VL and COVID reviews, and a 35.7-rating optimal threshold for the AB review. Llama 3.1 8B had 65-, 97- and 55-rating optimal thresholds for the VL, AB, and COVID-19 review, respectively. Optimal threshold ratings for Gemma 2 9B models were 75 (VL), 55 (AB), and 35 (COVID-19).

When using optimal rating thresholds and title/abstract screening as a reference (Table 3), balanced accuracy ranged from 76.7% to 91.3% across the three systematic reviews. Sensitivity was higher than 80% across all the three reviews and specificity ranged from 70.4% to 92.7%. Workload saving showed a pattern similar to specificity, ranging from 69.3% to 91.8%. PPVs were low for all models across the three reviews, not exceeding 17.7%, while NPVs were always above 99%. Overall, the COVID-19 review showed the highest balanced accuracy, sensitivity, and specificity values for each of the three models (89.1%–91.3% balanced accuracy, 87.5%–93.1% sensitivity, and 88.0%–92.7% specificity).

**Table 3:** LLM performance metrics, expressed as percentage (%), by systematic review

		Records screened by title/abstract used as a reference						
Systematic		Optimal rating						
review	LLM	threshold	bAcc	Sens	Spec	PPV	NPV	WS
VL	GPT-4o mini	47.5	87.0	93.8	80.3	15.3	99.7	77.4
	Llama 3.1 8B	65.0	84.1	82.8	85.4	17.7	99.2	82.3
	Gemma 2 9B	75.0	86.0	87.5	84.6	17.7	99.4	81.5
AB	GPT-4o mini	37.5	83.8	91.9	75.7	5.5	99.8	74.5
	Llama 3.1 8B	97.0	76.7	82.9	70.4	4.2	99.6	69.3
	Gemma 2 9B	55.0	84.9	89.8	80.0	6.5	99.8	78.8
COVID-19	GPT–4o mini	47.5	90.1	87.5	92.7	10.2	99.9	91.8
	Llama 3.1 8B	55.0	91.3	93.1	89.6	7.8	99.9	88.7
	Gemma 2 9B	35.0	89.1	90.3	88.0	6.6	99.9	87.1
		Records screened by full-text used as a reference						
Systematic		Optimal rating						
review	LLM	threshold	bAcc	Sens	Spec	PPV	NPV	WS
VL	GPT-4o mini	47.5	89.2	100.0	78.4	4.6	100.0	77.6
	Llama 3.1 8B	65.0	89.1	94.4	83.7	5.7	99.9	82.9
	Gemma 2 9B	75.0	85.8	88.9	82.7	5.0	99.9	81.8
AB	GPT-4o mini	37.5	85.4	96.0	74.7	0.4	100.0	74.7
	Llama 3.1 8B	97.0	82.8	96.0	69.7	0.4	100.0	69.6
	Gemma 2 9B	55.0	89.5	100.0	79.0	0.6	100.0	78.9
COVID-19	GPT–4o mini	47.5	94.8	97.3	92.4	5.8	100.0	92.0
	Llama 3.1 8B	55.0	94.6	100.0	89.2	4.3	100.0	88.8
	Gemma 2 9B	35.0	91.1	94.6	87.6	3.6	100.0	87.2

*Note:* Optimal rating thresholds were used.

LLM: large language model; bAcc: balanced accuracy; Sens: sensitivity; Spec: specificity; PPV: positive predictive value; NPV: negative predictive value; WS: workload saving; VL: vaccine literacy; AB: *A. baumannii*; COVID-19: coronavirus disease 2019; GPT: Generative Pretrained Transformer.

Using full-text screening as a reference, balanced accuracy rose to 82.8%–94.8%, as well as the sensitivity, with at least one model per review achieving perfect sensitivity (GPT-4o mini for the VL review, Gemma 2 9B for the AB review, and Llama 3.1 8B for the COVID-19 review), while specificity went down slightly. NPV values were consistently above 99%, but lower PPVs were reached (0.4%–5.8%).

Finally, the optimal rating thresholds for different models achieved a performance similar to the majority voting approach with a 50-rating threshold for all reviews.

### 3.4 Invalid responses, requests per minute, overall time to responses, and costs

GPT-4o mini generated no invalid responses across any of the systematic reviews (Table 4). However, low proportions of invalid responses were observed for Llama 3.1 8B and Gemma 2 9B (0.1%–0.3%). GPT-4o mini had the highest responses per minute rate (500 per minute), leading to the shortest overall time to responses (~3 minutes for VL, ~42 minutes for AB, and ~ 15 minutes for COVID-19 systematic reviews, respectively). Llama 3.1 8B had a consistent response rate of ~110–118 per minute across all reviews, with the overall time notably longer, especially for the AB review (~3 hours 11 minutes). Gemma 2 9B had the slowest response rate (~82–87 per minute) and the longest times across all three reviews, reaching the longest observed overall time in the AB review (~4 hours 15 minutes). Finally, overall costs for using GPT-4o mini varied between 0.14 and .93 USD per review, while Llama 3.1 8B and Gemma 2 9B were free to use.

**Table 4:** Characteristics of LLMs, invalid responses, responses per minute, overall time to responses and overall costs, by LLM and systematic review

		Context window,		Invalid responses,	Responses per minute,	Overall time	
LLM	*N* parameters	*N* tokens	Systematic review	*N* (%)	*N*/min	to responses	Overall costs
GPT-4o mini	Not disclosed	128,000	VL	0 (0.0)	500/min^a^	~3 min^a^	~0.14 USD^a^
			AB	0 (0.0)	~42 min^a^	~1.93 USD^a^
			COVID-19	0 (0.0)	~15 min^a^	~1.02 USD^a^
Llama 3.1 8B	8 billion	128,000	VL	5 (0.3)	~117/min^b^	~14 min^b^	Free
			AB	3 (0.0)	~110/min^b^	~3 h 11 min^b^	
			COVID-19	7 (0.1)	~118/min^b^	~1 h 4 min^b^	
Gemma 2 9B	9 billion	8,192	VL	5 (0.3)	~83/min^b^	~20 min^b^	Free
			AB	19 (0.1)	~82/min^b^	~4 h 15 min^b^	
			COVID-19	10 (0.1)	~87/min^b^	~1 h 28 min^b^	

*Note:* LLM: large language model; GPT: generative pretrained transformer; VL: vaccine literacy; AB: *Acinetobacter baumannii*; COVID-19: coronavirus disease 2019; USD: United States dollar.

a GPT-4o mini has a fixed rate of 500 requests per minute and a limitation of 10,000 requests per day maximum^47^ and a pricing of 0.15 USD/million input tokens and 0.6 USD/million output tokens on API usage tier 1^46^.

b Requests per minute and overall times are based on computations run on the local machine.

## 4 Discussion

In the selection process of articles for systematic review articles, the primary concern is the completeness of results, meaning that all relevant articles should be included.^44^ ^,^ ^45^ In other words, the cost of excluding relevant articles (i.e., producing false negatives) is generally considered higher than including irrelevant ones (i.e., producing false positives). Therefore, we believe that the sensitivity of a model should be prioritized, while its imprecision can be tolerated. The results of our study align with these *desiderata*, showing that, at least for the reviews under examination, the LLMs used are highly sensitive tools for the screening of citations, capable of achieving perfect sensitivity for including relevant records (i.e., full-text documents). Conversely, LLMs were also found to be highly imprecise, tending to overinclude irrelevant records. In addition, it is essential that high sensitivity is accompanied by reasonable specificity, and consequently, by significant workload savings. In line with this, we observed sufficient specificities and high NPVs, indicating that the models are much better at excluding irrelevant articles, which implies significant workload savings. These results may be partly due to our prompt design, which was aimed to maximize the models’ sensitivity, and partly to the typical class imbalance in systematic reviews, where the proportion of included articles is low, as observed in Syriani’s^11^ ^,^ ^12^ and Sanghera’s^26^ experiments. However, performances characterized by high sensitivity, specificity, NPV, and low precision are consistent with similar studies that employed multiple models^17^ or different versions of GPT only.^11^ ^,^ ^12^ In addition, no single model consistently outperformed the others across individual reviews; some performed better in certain cases, while the majority voting approach provided more balanced performance. Thus, combining multiple models’ decisions could help overcome the limitations of a single model.^38^

In parallel with performance, practical aspects of LLM usage should be considered. In this study, GPT-4o mini, presented as the most cost-effective OpenAI model accessible via API,^27^ ^,^ ^46^ was the fastest among the tested models, with a total cost of approximately 3 USD (Table 4). However, it is important to note that OpenAI API usage as a tier 1 user imposes a daily limit of 10,000 requests,^47^ which restricts the number of records that can be screened in one day. For very large corpora, this limitation requires splitting requests over several days or sending requests in batch.^48^ Another drawback is that API usage requires an internet connection, which may imply stability issues. Moreover, proprietary models, like those by OpenAI, do not fully disclose their characteristics and are subject to updates and deprecations,^49^ which can severely hinder the reproducibility of results.^50^ On the other hand, Llama 3.1 8B and Gemma 2 9B performed screening less quickly than GPT-4o mini, but certainly faster than a human reviewer. In addition, these open-source models can be run on conventional local machines, although they have minimum system requirements,^51^ and processing times are significantly influenced by hardware availability, such as the presence of a compatible GPU.^52^

This study has strengths and limitations. First, this study has explored the potential of compact LLMs for title and abstract screening in systematic reviews, including models that can be run on conventional local machines. In contrast, most existing studies have focused primarily on larger, noncompact GPT models from OpenAI.^39^ ^,^ ^53^ ^,^ ^54^ Likewise, one of the main strengths is the practical approach, aimed at not only assessing the performance of LLMs, but also processing time and costs, which may be a major bottleneck for their usage, especially in resource-limited settings. Moreover, we tried to mitigate the retrospective nature of the automated title/abstract screening evaluation,^55^ by using predetermined 25-, 50- and 75-rating thresholds, along with a majority-voting ensemble strategy to combine different models’ decisions. This approach achieved performance results comparable to those obtained using optimal thresholds, with the 50- and 75-rating thresholds serving as reasonable proxies for optimal thresholds in the explored systematic reviews. Indeed, when adopting a prospective approach, an optimal threshold is unknown, and it is likely that different models have different optimal rating thresholds. Using a 50-rating threshold with a majority voting method may be a reasonable option to assess LLMs’ performance in a prospective setting. Third, the reviews under consideration were diverse by topic, inclusion criteria, and size, and the LLM could be flexibly adapted to different contexts, while still reducing workloads and identifying relevant articles. In this regard, we observed that the COVID-19 review exhibited the highest sensitivity and specificity values across all models, compared to the other reviews. This may be since randomized controlled trials (RCTs) were an inclusion criterion in the COVID-19 review, and RCTs typically have stricter reporting standards and a more structured abstract format, compared to studies with different designs.^56^ In relation to this, the agreement on inclusion between models at different thresholds could be used as a way to quantify the quality of abstract writing—that is, if similar accuracy is achieved across different rating thresholds, it may indicate high overall abstract clarity.

On the other hand, limitations must be acknowledged. First, the small number of systematic reviews considered restricts the generalizability of our findings, and they should be interpreted with caution. Indeed, the limited number of reviews may have introduced a potential selection bias, as the specific reviews we analyzed do not fully represent the spectrum of available literature. A broader or different sample of reviews, spanning a wider range of disciplines and topics, may have achieved different conclusions. Nonetheless, in our view, these results are promising and informative, as the reviews we selected were intentionally diverse in research question, study design involved, and complexity, contributing to the ever-growing evidence in this area. As a second limitation, the choice of compact LLMs, with a reduced number of parameters, was primarily driven by cost, time, and hardware considerations, and LLMs with a higher number of parameters may achieve better results.^20^ On a similar note, we adopted a zero-shot prompting strategy, which is considered the simplest and most conservative.^11^ ^,^ ^12^ However, as noted in other studies, model performance heavily depends on the type of prompt used, and, although there is no universal approach to prompt optimization,^25^ it is possible that different prompt engineering approaches could yield better performance. Moreover, the majority of records across reviews were published before the LLMs’ knowledge cut-off point—i.e., the end of their training was disclosed. This raises the possibility that the models were trained on these records, potentially influencing our findings, as transparency in training sources is often lacking.^57^ A prospective approach to title and abstract screening could help clarify the impact of different knowledge cut-offs on the models’ performance. In addition, as the agreement between human reviewers in the original screening was not recorded, we could not compare LLMs with human reviewers and, thus, assumed the human screening to be the ground truth. However, other studies^11^ ^,^ ^12^ found that in corpora with a low proportion of included records and a low proportion of decision conflicts between human reviewers, LLMs tend to show high sensitivity and low precision, as observed in our case, which may indirectly indicate a high level of agreement between human reviewers, although a certain degree of disagreement on false positives between human reviewers and LLMs should be expected.

## 5 Conclusions

In light of this and other studies,^15^ ^,^ ^16^ ^,^ ^39^ from a technical standpoint, LLMs can feasibly be employed for screening records by title and abstract in systematic reviews. However, the Cochrane Collaboration underlines the need for validation of LLMs in systematic reviews.^1^ As suggested in other works,^15^ ^,^ ^39^ a potential application of LLMs for title and abstract screening, especially when the number of identified records is extremely large, appears to be as a first-screener or triage tool. In this role, the model performs an initial screening of titles and abstracts, leaving the human reviewers with the records included by the model for full-text screening. This approach may be very convenient in the context of rapid reviews, where balancing workload reduction and completeness is crucial.^58^ ^,^ ^59^ However, for this approach to be fully reliable, LLMs must show perfect sensitivity—otherwise, relevant records may be permanently missed. Another, more conservative, approach is to use LLMs as a second-screener for title and abstract screening—that is, to combine the model’s decisions with those of human reviewers either “in parallel” (i.e., inclusion results from either the LLM’s or the human’s decision) or “in series” (i.e., inclusion requires agreement between both the LLM and the human).^26^ These two schemata increase overall sensitivity or precision at each other’s expense, respectively. Rating thresholds may further refine this trade-off, with lower thresholds allowing for higher sensitivity and higher thresholds improving precision. Moreover, as the volume of published literature continues to grow, the workload associated with screening in systematic reviews is expected to rise significantly,^60^ with title and abstract screening, already one of the most error-prone stages of the systematic review process^61^ becoming increasingly susceptible to mistakes as the number of records expands.^62^ In our view, given the need to preserve sensitivity, combining human and LLM decisions “in parallel” may be the most reasonable way to integrate these models into the systematic review workflow, as this approach may rescue potentially relevant studies overlooked by the human reviewer, enhance confidence in excluding irrelevant records, when both agree on exclusions, and ultimately increase sensitivity. In contrast, with an “in series” combination of decisions, relevant records may be lost due to errors from either the human reviewer or the LLM—or, at least, conflicting decisions between the human screener and LLM should be resolved by a third human opinion. Nevertheless, to ensure the safe and effective integration of LLMs into systematic review workflows, further investigation is essential, particularly through studies adopting a prospective approach to the assessment of title and abstract screening.

## Supporting information

Sciurti et al. supplementary material Sciurti et al. supplementary material

## Data Availability

Datasets and R code used in this research are available under a CC-BY-4.0 license on Open Science Framework (OSF): https://osf.io/kjnwt.

## Supplementary material

To view supplementary material for this article, please visit http://doi.org/10.1017/rsm.2025.10044.
