# Supplementary material for: Compact large language models for title and abstract screening in systematic reviews: An assessment of feasibility, accuracy, and workload reduction
Source: Res Synth Methods. 2025 Nov 13;17(2):332–47. doi: 10.1017/rsm.2025.10044 (PMC12873614; doi:10.1017/rsm.2025.10044)
Supplement: Sciurti et al. supplementary material [file S1759287925100446sup001.pdf]

Supplementary Material

Table of Contents

Table S1. Page 1
Table S2. Page 2
Table S3. Page 3

Table S1. Examples of prompts, by systematic review.

VL

Context	###<br>You are an experienced systematic review author. You are screening records for a systematic review about the association between vaccine literacy and vaccination intention or status.
Instructions	You have the records' **title** and **abstract** as input. Check the record's title and abstract and rate it, in order to decide if it should be included for further assessment and full-text screening, according to your inclusion criteria. Use the inclusion criteria below to make your decision:<br>- Criterion A. Records must explore vaccine literacy explicitly.<br>- Criterion B. Records must explore vaccination intention or status.<br>Based on how confident you are that the record should be included, give the record a rating from 0 (least confident) to 100 (most confident). Answer with a rating number ranging from 0 to 100 in an integer form only. No further explanation is needed.<br>###
Task	###<br>**title**: {What Does COVID-19 Vaccine Efficacy Really Mean? Interpreting Clinical Trial Results in the Context of Relative and Absolute Risk Reduction}<br>**abstract**: {With the introduction of several COVID-19 vaccine candidates, vaccine efficacy has been widely reported; however, the meaning of vaccine efficacy is often unclear. The purpose of this commentary is to explain how vaccine efficacy is determined in the context of relative and absolute risk reduction. An illustrative example is provided using the results of the adolescent Pfizer-Biontech COVID-19 vaccine trial. We suggest guidelines for interpreting and communicating clinical trial evidence to help Certified Health Education Specialists (CHES®), Master Certified Health Education Specialists (MCHES®), and other health education professionals enhance COVID-19 vaccine literacy in the general public.}<br>###

AB

Context	###<br>You are an experienced systematic review author. You are screening records for a systematic review about the association between the use of antimicrobials and infection or colonization by antimicrobial-resistant Acinetobacter baumannii in patients hospitalized in Intensive Care Units (ICUs).
Instructions	You have the records' **title** and **abstract** as input. Check the record's title and abstract and rate it, in order to decide if it should be included for further assessment and full-text screening, according to your inclusion criteria.<br>Use the inclusion criteria below to make your decision:<br>- Criterion A. Records must be observational studies (e.g. cohort or case-control studies), considering adult patients only, hospitalized in an ICU setting.<br>- Criterion B. Records must explore infection or colonization by Acinetobacter baumannii resistant to any antimicrobial agent or class of antimicrobial agents.<br>- Criterion C. Records must explore the use of any antimicrobial agent or class of antimicrobial agents.<br>Based on how confident you are that the record should be included, give the record a rating from 1 (least confident) to 100 (most confident). Answer with a rating number ranging from 1 to 100 in an integer form only. No further explanation is needed.<br>###
Task	###<br>**title**: {Three-dimensional modified test and analysis of the present status of β-lactamases and resistance of the clinical isolates of Acinetobacter baumannii}<br>**abstract**: {Objective: To understand the present status of β-lactamases and resistance to 12 common antimicrobial agents in clinical isolates of Acinetobacter baumannii. Methods: The β-lactamases production was detected early by the cefinase slip method to 120 Acinetobacter baumannii strains from clinical specimens in our hospital. The types of β-lactamases production were distinguished by a three-dimensional modified test using a big agar plates in those isolates. The MICs of 12 common antimicrobial agents to these isolates were determined by 2-fold agar dilution method followed by CLSI recommendations. Results: Except IMP, the resistant rates of 120 isolates to 11 common antimicrobial agents were more than 50%. There were 114 strains producing β-lactamases (95%), in which, 63 isolates were typed by the three-dimensional modified test using a big agar plates, there were 4 strains (3.51%), 15 strains (13.16%), 5 strains (4.39%) and 18 strains (15.78%) producing carbapenemases, ESBLs, AmpC enzyme, and IRTs, respectively; there were 5 strains (4.39%), 9 strains (7.89%) and 7 strains (6.14%) producing carbapenemases plus ESBLs, AmpC, ESBLs, and IRTs, respectively. Conclusion: Results showed that the three-dimensional modified test with a big agar plates was an effective approach for the detection and differentiation of various multiplex types of β-lactamases.}<br>###

COVID-19

Context	###<br>You are an experienced systematic review author. You are screening records for a systematic review about vitamin supplementation for prevention of SARS-CoV-2 infections and/or management of patients with COVID-19 or long-COVID.
Instructions	You have the records' **title** and **abstract** as input. Check the record's title and abstract and rate it, in order to decide if it should be included for further assessment and full-text screening, according to your inclusion criteria.<br>Use the inclusion criteria below to make your decision:<br>- Criterion A. Records must be Randomized Controlled Trials (RCTs).<br>- Criterion B. Records must explore any vitamin used to prevent SARS-CoV-2 infections or treat patients with COVID-19 or long-COVID, compared to placebo or other treatments. Consider any of the following vitamins: 'vitamin A', 'carotenoids', 'beta carotene', 'β carotene', 'carotene', 'retinol', 'vitamin D', 'vitamin D2', 'vitamin D3', 'ergocalciferol', 'paricalcitol', 'cholecalciferol', 'calcitriol', 'calcifediol', 'vitamin E', 'tocopherol', 'alpha tocopherol', 'beta tocopherol', 'gamma tocopherol', 'tocotrienol', 'vitamin K', 'vitamin K1', 'phytonadione', 'vitamin K2', 'menaquinone', 'vitamin K3', 'menadione', 'vitamin B', 'B complex', 'vitamin B1', 'thiamine', 'vitamin B2', 'riboflavin', 'vitamin G', 'vitamin B3', 'niacin', 'niacinamide', 'nicotinic acid', 'vitamin PP', 'vitamin B5', 'pantothenic acid', 'pantothenate', 'vitamin B6', 'pyridoxine', 'pyridoxal', 'pyridoxamine', 'vitamin B7', 'biotin', 'vitamin H', 'vitamin B9', 'folate', 'folic acid', 'l-methylfolate', 'vitamin M', 'vitamin B12', 'cyanocobalamin', 'methylcobalamin', 'hydroxocobalamin', 'vitamin C', 'ascorbic acid', 'ascorbate'.<br>Based on how confident you are that the record should be included, give the record a rating from 0 (least confident) to 100 (most confident). Answer with a rating number ranging from 0 to 100 in an integer form only. No further explanation is needed.<br>###
Task	###<br>**title**: {Oxidative stress status in covid-19 patients hospitalized in intensive care unit for severe pneumonia. A pilot study}<br>**abstract**: {Background: A key role of oxidative stress has been highlighted in the pathogenesis of COVID-19. However, little has been said about oxidative stress status (OSS) of COVID-19 patients hospitalized in intensive care unit (ICU). Material and Methods: Biomarkers of the systemic OSS included antioxidants (9 assays), trace elements (3 assays), inflammation markers (4 assays) and oxidative damage to lipids (3 assays). Results: Blood samples were drawn after 9 (7–11) and 41 (39–43) days of ICU stay, respectively in 3 and 6 patients. Vitamin C, thiol proteins, reduced glutathione, γ-tocopherol, β-carotene and PAOT® score were significantly decreased compared to laboratory reference values. Selenium concentration was at the limit of the lower reference value. By contrast, the copper/zinc ratio (as a source of oxidative stress) was higher than reference values in 55% of patients while copper was significantly correlated with lipid peroxides (r = 0.95, p < 0.001). Inflammatory biomarkers (C-reactive protein and myeloperoxidase) were significantly increased when compared to normals. Conclusions: The systemic OSS was strongly altered in critically ill COVID-19 patients as evidenced by increased lipid peroxidation but also by deficits in some antioxidants (vitamin C, glutathione, thiol proteins) and trace elements (selenium).}<br>###

VL: Vaccine Literacy; AB: *Acinetobacter baumannii*; COVID-19: Coronavirus Disease 2019.

Table S2. Formal description of performance metrics.

Performance metric	Mathematical definition	Definition
Sensitivity (recall)	$\frac{TP}{TP + FN}$	The proportion of all actually included records that were correctly included by the model.
Specificity	$\frac{TN}{TN + FP}$	The proportion of all actually excluded records that were correctly excluded by the model.
Balanced accuracy	$\frac{Sensitivity + Specificity}{2}$	The arithmetic mean of sensitivity and specificity.
PPV (precision)	$\frac{TP}{TP + FP}$	The proportion of all the records included by the model that were actually included.
NPV	$\frac{TN}{TN + FN}$	The proportion of all the records excluded by the model that were actually excluded.
Workload saving	$\frac{TN}{TP + TN + FP + FN}$	The proportion of actually excluded records.

TP: True Positives; TN: True Negatives; FP: False Positives; FN: False Negatives. PPV: Positive Predictive Value; NPV: Negative Predictive Value.

Table S3. TRIPOD + LLM checklist for studies evaluating LLMs in classification tasks.

Section/Topic	Item number	Checklist Item	Research Design	LLM Task	Reported on page
**Title**	1	Identify the study as developing, fine-tuning, and/or evaluating the performance of an LLM, specifying the task, the target population, and the outcome to be predicted.	All	All	1
**Abstract**					
Background	2b	Provide a brief explanation of the healthcare context, use case and rationale for developing or evaluating the performance of an LLM.	E, H	All	1
Objectives	2c	Specify the study objectives, including whether the study describes LLMs development, tuning, and/or evaluation	All	All	1
	2d	Describe the key elements of the study setting.	All	All	1
	2e	Detail all data used in the study, specify data splits and any selective use of data.	M, D, E	All	1
	2f	Specify the name and version of LLM used.	All	All	3
Methods	2g	Briefly summarize the LLM-building steps, including any fine-tuning, reward modeling, reinforcement learning with human feedback (RLHF), etc.	M, D	All	N/A
	2h	Describe the specific tasks performed by the LLMs (e.g., medical QA, summarization, extraction), highlighting key inputs and outputs used in the final LLM.	All	All	1
	2i	Specify the evaluation datasets/populations used, including the endpoint evaluated, and detail whether this information was held out during training/tuning where relevant, and what measure(s) were used to evaluate LLM performance.	All	All	1
Results	2j	Give an overall report and interpretation of the main results.	All	All	1
Discussion	2k	Explicitly state any broader implications or concerns that have arisen in light of these results.	All	All	1
Other	2l	Give the registration number and name of the registry or repository (if relevant).	H	All	N/A
**Introduction**					
Background	3a	Explain the healthcare context / use case (e.g., administrative, diagnostic, therapeutic, clinical workflow) and rationale for developing or evaluating the LLM, including references to existing approaches and models.	All	All	2
	3b	Describe the target population and the intended use of the LLM in the context of the care pathway, including its intended users in current gold standard practices (e.g., healthcare professionals, patients, public, or administrators).	E, H	All	2
Objectives	4	Specify the study objectives, including whether the study describes the initial development, fine-tuning, or validation of an LLM (or multiple stages).	All	All	2
**Methods**					
Data	5a	Describe the sources of data separately for the training, tuning, and/or evaluation datasets and the rationale for using these data (e.g., web corpora, clinical research/trial data, EHR data).	All	All	2-3
	5b	Describe the relevant data points and provide a quantitative and qualitative description of their distribution and other relevant descriptors of the dataset (e.g., source, languages, countries of origin)	All	All	3
	5c	Specifically state the date of the oldest and newest item of text used in the development process (training, fine-tuning, reward modeling) and in the evaluation datasets.	M, D, E, H	All	3, Table 1
	5d	Describe any data pre-processing and quality checking, including whether this was similar across text corpora, institutions, and relevant sociodemographic groups.	All	All	N/A
	5e	Describe how missing and imbalanced data were handled and provide reasons for omitting any data.	M, D, E	All	N/A
Analytical Methods	6a	Report the LLM name, version, and last date of training or use during inference.	All	All	3
	6b	Specify the type of LLM architecture, and LLM building steps, including any hyperparameter tuning (e.g., temperature, length limits, penalties), prompt engineering, and any inference settings (e.g., seed, temperature, max token length) as relevant.	M, D, E	All	7-8
	6c	Report details of LLM development process from text input to outcome generation, such as training, fine-tuning procedures, and alignment strategy (e.g., reinforcement learning, direct preference optimization, etc.) and alignment goals (e.g., helpfulness, honesty, harmlessness, etc.).	M, D	All	N/A
	6d	Specify the initial and post-processed output of the LLM (e.g., probabilities, classification, unstructured text).	All	All	3

	6e	Provide details and rationale for any classification and how the probabilities were determined and thresholds identified.	All	C, OF	3
	6f	Include metrics that capture the quality of generative outputs, such as consistency, relevance, and accuracy, compared to gold standards.	All	QA, IR, DG, SS, MT	N/A
	6g	Report the outcome metrics' relevance to downstream task at deployment time and correlation of metric to human evaluation of the text for the intended use.	E, H	All	3-4
LLM Output	7a	Clearly define the outcome, how the LLM predictions were calculated (e.g., formula, code, object, API), and evaluation metrics.	E, H	All	3-4
	7b	If outcome assessment requires subjective interpretation, describe the qualifications of the assessors, any instructions provided, relevant information on demographics of the assessors, and inter-assessor agreement.	All	All	N/A
	7c	Specify how performance was compared to other LLMs, humans, and other benchmarks or standards.	All	All	4
Annotation	8a	If annotation was done, report how text was labeled, including providing specific annotation guidelines with examples.	All	All	3
	8b	If annotation was done, report how many annotators labeled the dataset(s), including the proportion of data in each dataset that were annotated by more than 1 annotator.	All	All	3
	8c	If annotation was done, provide information on the background and experience of the annotators, and the inter-annotator agreement.	All	All	3
Prompting	9a	If research involved prompting LLMs, provide details on the processes used during prompt design, curation, and selection.	All	All	3, Table S1
	9b	If research involved prompting LLMs, report what data were used to develop the prompts.	All	All	6
Summarization	10	Describe any preprocessing of the data before summarization.	All	SS	N/A
Instruction Tuning / Alignment	11	If instruction tuning/alignment strategies were used, what were the instructions and interface used for evaluation, and what were the characteristics of the populations doing evaluation?	M, D	All	N/A
Compute	12	Report compute, or proxies thereof (e.g., time on what and how many machines, cost on what and how many machines, inference time, floating-point operations per second (FLOPs)), required to carry out methods.	M, D, E	All	3, Table 4
Ethics Approval	13	Name the institutional research board or ethics committee that approved the study and describe the participant-informed consent or the ethics committee waiver of informed consent.	All	All	N/A
Open Science	14a	Give the source of funding and the role of the funders for the present study.	All	All	N/A
	14b	Declare any conflicts of interest and financial disclosures for all authors.	All	All	N/A
	14c	Indicate where the study protocol can be accessed or state that a protocol was not prepared.	H	All	N/A
	14d	Provide registration information for the study, including register name and registration number, or state that the study was not registered.	H	All	N/A
	14e	Provide details of the availability of the study data.	All	All	3, 5
	14f	Provide details of the availability of the code to reproduce the study results.	All	All	3, 5
Public Involvement	15	Provide details of any patient and public involvement during the design, conduct, reporting, interpretation, or dissemination of the study or state no involvement.	H	All	N/A
**Results**					
Participants	16a	When using patient/EHR data, describe the flow of text/EHR/patient data through the study, including the number of documents/questions/participants with and without the outcome/label and follow-up time.	E, H	All	N/A
	16b	When using patient/EHR data, report the characteristics overall and, for each data source or setting, and for development/evaluation splits, including the key dates, key predictors, and sample size.	E, H	All	N/A
	16c	For LLM evaluation, show a comparison of the distribution of important predictors between development and evaluation data.	E, H	All	N/A
	16d	When using patient/EHR data, specify the number of participants and outcome events in each analysis (e.g., for LLM development, hyperparameter tuning, LLM evaluation).	E, H	All	N/A
Performance	17	Report LLM performance according to pre-specified metrics (see item 7a) and/or human evaluation (see item 7d).	All	All	5-10, Table 2-3
LLM Updating	18	If applicable, report the results from any LLM updating, including the updated LLM and subsequent performance.	All	All	N/A

**Discussion**					
Interpretation	19a	Give an overall interpretation of the main results, including issues of fairness in the context of the objectives and previous studies.	All	All	10
Limitations	19b	Discuss any limitations of the study and their effects on any biases, statistical uncertainty, and generalizability.	All	All	12-13
Usability of the LLM in context	19c	Describe any known challenges in using data for the specified task and domain context with reference to representation, missingness, harmonization, and bias.	E, H	All	11-12
	19d	Define the intended use for the implementation under evaluation, including the intended input, end-user, level of autonomy/human oversight.	E, H	All	13-14
	19e	If applicable, describe how poor quality or unavailable input data should be assessed and handled when implementing the LLM, i.e., what is the usability of the LLM in the context of current clinical care.	E, H	All	13-14
	19f	If applicable, specify whether users will be required to interact in the handling of the input data or use of the LLM, and what level of expertise is required of users.	E, H	All	N/A
	19g	Discuss any next steps for future research, with a specific view to applicability and generalizability of the LLM.	All	All	13

TRIPOD: Transparent Reporting of a multivariable model for Individual Prognosis Or Diagnosis; LLM: Large Language Model; M: LLM methods; D: *de novo* LLM development; E: LLM evaluation; H: LLM evaluation in healthcare settings; C: classification; OF: outcome forecasting; QA: long-form question answering; IR: information retrieval; DG: document generation; SS: summarization and simplification; MT: machine translation; API: application programming interface; N/A: Not applicable.
